# Comparative evaluation of porcine and bovine bone xenografts in bone grafting: a systematic review and meta-analysis

**DOI:** 10.1186/s40729-025-00630-w

**Published:** 2025-06-09

**Authors:** Kun Wang, Jiatong Zhang, Mengyao Ding, Yifan Xie, Yan Wang, Chuyi Jin, Mengqing Yan, Lipei Liu, Cheng Ding, Xing Chen

**Affiliations:** 1 Stomatology Center, Affiliated Hospital of Hangzhou Normal University, Hangzhou, Zhejiang Province 310015 China; 2 School of Stomatology, Hangzhou Normal University, Hangzhou, Zhejiang Province China

**Keywords:** Bone substitutes, Maxillary sinus floor augmentation, Alveolar ridge preservation, Xenograft

## Abstract

**Objective:**

To compare the efficacy of porcine bone xenografts (PBX) and bovine bone xenografts (BBX) in maxillary sinus floor augmentation (MSFA) and alveolar ridge preservation (ARP), focusing on histomorphometric and radiographic outcomes.

**Materials and methods:**

A comprehensive online search for relevant studies was conducted using PubMed, Cochrane Library, Web of Science, and Scopus, including literature published up to April 2025 (PROSPERO CRD42024628683). The percentage of newly formed bone (NFB) served as the primary outcome. Secondary outcomes included histomorphometric results such as residual bone graft (RBG) and connective tissue (CT), as well as radiographic results, including vertical height reduction, horizontal width reduction, and volume reduction.

**Results:**

Out of 577 initially identified records, 10 studies were included. The analysis included 239 sites grafted with BBX (51 in MSFA, 188 in ARP) and 213 sites with PBX (51 in MSFA, 162 in ARP). A total of 6 studies reported data on NFB across 202 grafted sites (101 PBX, 101 BBX). The meta-analysis found no significant difference in NFB between PBX and BBX (WMD = 1.5, 95% CI: −1.46 to 4.46; *p* = 0.321), with moderate heterogeneity (I² = 44.6%). For each secondary outcome, no statistically significant difference was shown between the two groups.

**Conclusion:**

This systematic review found no significant differences in histomorphometric and radiographic outcomes between PBX and BBX in bone grafting (MSFA/ARP), supporting the clinical comparability of PBX as an alternative to BBX. Further well-designed randomized controlled trials are needed to evaluate long-term outcomes such as implant survival, graft resorption, and bone stability.

**Clinical trial number:**

Not applicable.

## Introduction

Insufficient alveolar bone volume critically compromises the feasibility and long-term stability of dental implants. To address this challenge, alveolar ridge preservation (ARP) and maxillary sinus floor augmentation (MSFA)– as core techniques in modern bone augmentation– have emerged as foundational strategies in implant site development. These procedures aim to optimize osteogenic conditions within the recipient area and are recognized as clinically significant approaches with strong evidence supporting their utility in contemporary practice [1, 2]. When maxillary posterior teeth are lost, sinus pneumatization and alveolar bone atrophy often occur [3]. These changes reduce the available bone height, making implant placement more difficult. MSFA is a widely employed to ensure sufficient healthy bone mass and improve implant success by lifts the sinus floor and fills the space with bone graft material [4]. Similarly, tooth loss caused by severe periodontitis, the most prevalent chronic oral condition, can significantly disrupt alveolar structures [5, 6]. Post-extraction anatomical and functional alveolar bone loss poses significant challenges to achieving prosthodontically and aesthetically optimal implant outcomes. To address this issue, ARP aims to minimize bone loss and prevent excessive socket remodeling following tooth extraction [7]. Within these bony reconstruction protocols, bone substitute materials fulfill dual biological roles: serving as three-dimensional scaffolding for spatial maintenance while simultaneously initiating neo-osteogenesis through guided bone regeneration mechanisms. These synergistic effects ultimately establish the anatomical prerequisites for achieving prosthetically-driven three-dimensional implant positioning [8, 9].

Autogenous bone is widely regarded as the gold standard in bone grafting, given its immunologic compatibility and osteogenic, osteoinductive, and osteoconductive characteristics [10, 11]. However, autologous bone grafting is associated with significant disadvantages. For instance, a second surgical site may need to be opened, the incidence of complications could increase, and there may be an inevitable tendency for significant absorption [12]. Substitute materials offer significant advantages in dental procedures, including low surgical morbidity, an abundant supply of materials, and reduced surgery duration, as they do not require donor tissue harvest [13]. Of these materials, xenografts, particularly bovine bone xenograft (BBX), are widely used to bone regeneration in dental applications. BBX has been thoroughly tested in both preclinical and clinical studies, with strong evidence supporting its effectiveness in MSFA, guided bone regeneration (GBR), and ARP [14–16]. Despite its widespread use, concerns about bovine-specific disease transmission, as well as cultural and religious restrictions, have increased interest in porcine bone xenograft (PBX) as an alternative [17, 18]. Scarano, Piattelli [19] demonstrated that cortical porcine bone is both biocompatible and osteoconductive, making it a suitable option for MSFA without disrupting normal bone healing. Similarly, Orsini, Scarano [20] reported that PBX shares physicochemical properties with inorganic bovine bone, with only minor differences.

Since MSFA and ARP share key characteristics—both involving internal bone grafting within anatomically enclosed spaces such as the maxillary sinus or the extraction socket—they provide spatial containment and a stable healing environment. This enables more standardized surgical protocols and reproducible histomorphometric and radiographic evaluations. In contrast, other types of bone augmentation techniques, such as horizontal and vertical guided bone regeneration (GBR), vary widely in terms of surgical technique, defect morphology, membrane types, fixation methods, and postoperative healing protocols [21]. Based on both clinical and methodological considerations, including such procedures would have significantly increased methodological heterogeneity and limited the comparability of the data. Therefore, we deliberately narrowed the scope of this systematic review to focus solely on MSFA and ARP, which improves internal validity and allows for a more clinically meaningful comparison between PBX and BBX.

Several studies have compared PBX and BBX specifically in MSFA and ARP, but the current evidence remains limited and lacks comprehensive analysis of clinical outcomes. To address these gaps, this systematic review and meta-analysis focuses on MSFA and ARP procedures by evaluating the clinical performance of PBX relative to BBX.

## Materials and methods

This review protocol, developed following the principles outlined in the Cochrane guidelines and PRISMA recommendations [22]., was prospectively registered in the PROSPERO database (ID: CRD42024628683). This registration, completed before data analysis began, ensures adherence to prospective registration standards and enhances the transparency and reproducibility of the study.

### Objectives

This review aims to compare the efficacy of porcine bone xenografts (PBX) and bovine bone xenografts (BBX) in maxillary sinus floor augmentation (MSFA) and alveolar ridge preservation (ARP), focusing on histomorphometric and radiographic outcomes.

The PICO elements were as follows:

Patient: adult patients undergoing MSFA or ARP.

Intervention: bone grafted with PBX.

Comparison: bone grafted with BBX.

Outcome: Primary outcome: Percentage of newly formed bone (NFB).

Secondary outcomes: Other histomorphometric results, including residual bone graft (RBG) and connective tissue (CT); radiographic results, such as vertical height reduction (VHR), horizontal width reduction (HWR), and volume reduction (VR).

### Search strategy

A comprehensive online search for relevant studies was conducted using PubMed, Cochrane Library, Web of Science, and Scopus. The search had no language or publication year restrictions and was finalized in April 2025. Search strategies are reported in Table 1. To ensure comprehensive coverage, manual searches were conducted in major journals specializing in oral and maxillofacial surgery and implant dentistry. In addition, reference lists of included studies were screened to find potential articles.

**Table 1:** Search strategy

Database	Search Strategy
PubMed	((porcine bone) OR (porcine-derived bone) OR (deproteinized porcine bone) OR (porcine xenograft*)) AND ((bovine bone) OR (Bio-Oss) OR (deproteinized bovine bone) OR (bovine xenograft*)) AND ((ridge augmentation) OR (alveolar ridge preservation) OR (socket) OR (guided bone regeneration) OR (maxillary sinus floor augmentation) OR (maxillary sinus elevation) OR (maxillary sinus lift))
Cochrane Library	((porcine bone) OR (porcine-derived bone) OR (deproteinized porcine bone) OR (porcine xenograft*)) AND ((bovine bone) OR (Bio-Oss) OR (deproteinized bovine bone) OR (bovine xenograft*)) AND ((ridge augmentation) OR (alveolar ridge preservation) OR (socket) OR (guided bone regeneration) OR (maxillary sinus floor augmentation) OR (maxillary sinus elevation) OR (maxillary sinus lift))
Web of Science	TS=(((porcine bone) OR (porcine-derived bone) OR (deproteinized porcine bone) OR (porcine xenograft*)) AND ((bovine bone) OR (Bio-Oss) OR (deproteinized bovine bone) OR (bovine xenograft*)) AND ((ridge augmentation) OR (alveolar ridge preservation) OR (socket) OR (guided bone regeneration) OR (maxillary sinus floor augmentation) OR (maxillary sinus elevation) OR (maxillary sinus lift)))
Scopus	TITLE-ABS-KEY ((porcine bone) OR (porcine-derived bone) OR (deproteinized porcine bone) OR (porcine xenograft*) AND (bovine bone) OR (Bio-Oss) OR (deproteinized bovine bone) OR (bovine xenograft*) AND (ridge augmentation OR alveolar ridge preservation OR socket OR guided bone regeneration OR maxillary sinus floor augmentation OR maxillary sinus elevation OR maxillary sinus lift))

### Eligibility criteria

The inclusion criteria were as follows: (1) comparative studies directly evaluating PBX and BBX in MSFA or ARP; (2) studies involving human participants with quantitative outcomes, such as histomorphometric and/or radiographic measurements; (3) studies with a clearly defined control group to facilitate direct comparisons between the two materials. The analysis considered exclusively original research articles published in peer-reviewed journals. Studies focusing on animal models, review articles, conference abstracts, and those without sufficient outcome data were excluded.

### Data extraction

Relevant data and study characteristics were independently extracted from the selected articles by two reviewers (JCY and XYF), who also summarized the outcomes. Any disagreements were addressed through discussion with a third reviewer (WK).

### Risk of bias in studies and quality assessment

The risk of bias in the included RCTs was evaluated using the revised Cochrane risk-of-bias tool (ROB 2). The following five domains were assessed: randomization process, deviations from intended interventions, missing outcome data, outcome measurement, and selection of reported results. Each study was then classified as having low risk, some concerns, or high risk of bias [23].

For non-randomized studies, tools such as the Newcastle-Ottawa Scale (NOS) were applied, focusing on selection, cohort comparability, and the accuracy of exposure or outcome assessment. Studies were awarded stars, with a maximum of nine stars indicating the highest quality. Based on the star rating, studies were categorized into three groups: low quality (0–3 stars), moderate quality (4–6 stars), and high quality (7–9 stars) [24].

Two authors (WK and WY) independently assessed the methodological quality of the studies. Any discrepancies were resolved through discussion with a third reviewer (DMY).

### Data analysis

To accommodate variations in measurement tools and scales across studies, the mean difference (MD) and standardized mean difference (SMD) were employed as appropriate effect size indicators. Both were reported with 95% confidence interval (CI). The choice between fixed-effects and random-effects models was determined by the level of statistical heterogeneity, following the method described by DerSimonian and Laird [25]. Heterogeneity was assessed using both the Q statistic and I² index, with significance thresholds set at *P* < 0.10 or I² > 50% [26]. For cases where I² exceeded 50%, a one-way sensitivity analysis was conducted by sequentially excluding individual studies to evaluate their influence on the overall results. Publication bias was assessed using Egger’s regression test, with a significance threshold of *P* < 0.05. Effect sizes were visualized using forest plots, with the significance level set at 0.05 for all analyses. Statistical analyses were conducted using Stata 14.0 (StataCorp LP, College Station, TX, USA).

## Results

### Search

**Fig. 1:**
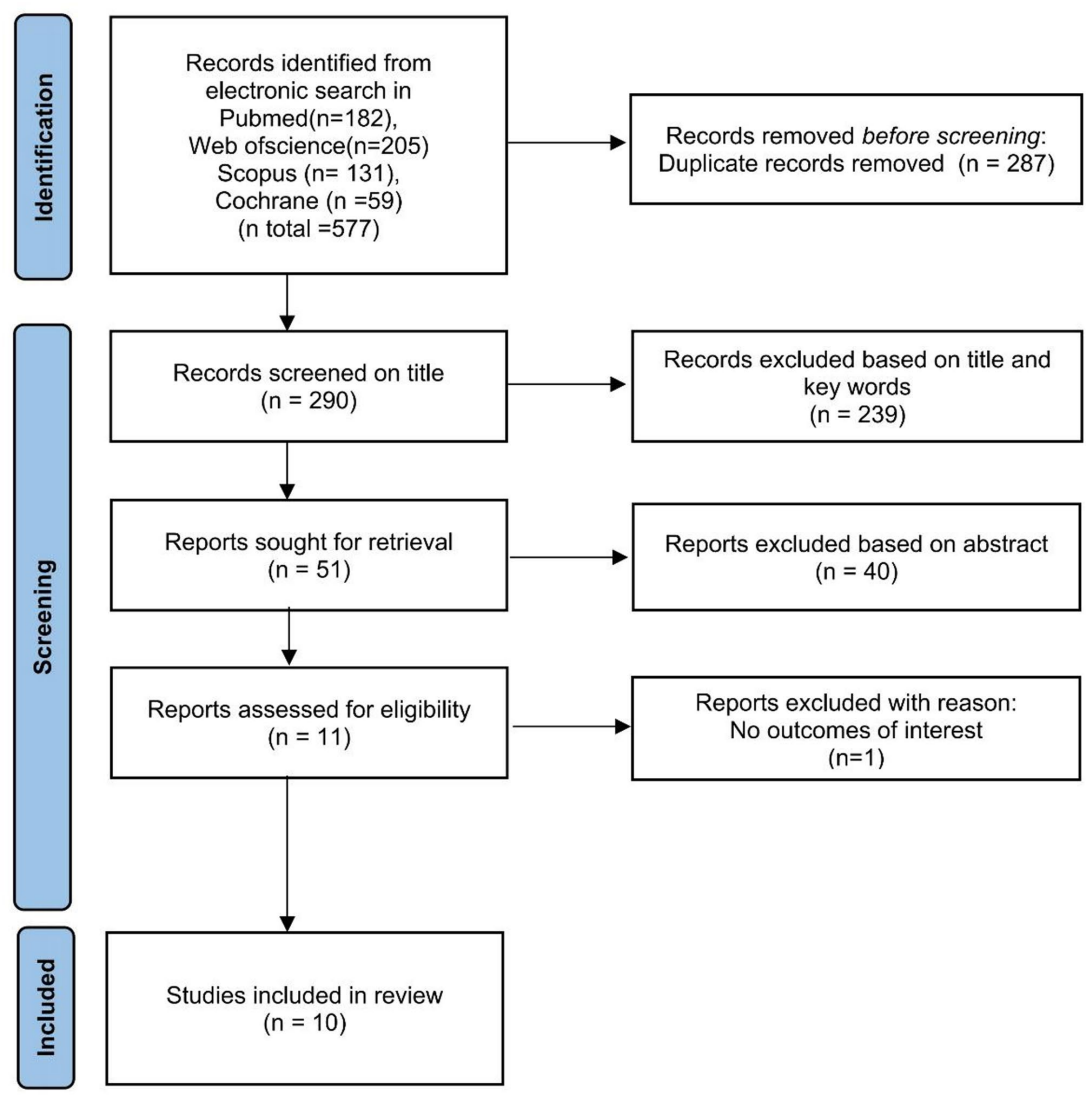
PRISMA flow chart on the search strategy

A total of 577 records were identified through the literature search. After removing duplicates, 287 records retained and were managed using Endnote 21 software. Following title and abstract screening, 279 records were excluded based on the inclusion and exclusion criteria, leaving 11 records for full-text review. Finally, 10 articles met the inclusion criteria for qualitative analysis [27–36]. No additional publications were found through manual screening of the reference lists in the selected studies. The study selection process is summarized in Fig. 1.

### Description of selected studies

The characteristics of qualified studies were shown in Table 2. This meta-analysis ultimately included 10 studies in total, comprising five studies on MSFA and five on ARP. Together, the studies reported on 452 grafted sites: 239 sites were treated with BBX, including 51 in MSFA and 188 in ARP, while 213 sites received PBX, including 51 in MSFA and 162 in ARP. Most of the studies included were RCTs, except the two by Iezzi, Degidi [35] and Lee, Kim [29], which were a non-randomized controlled clinical trial and a retrospective case–control study, respectively. Two of the selected studies had more than one experimental group [32, 35]. Among the eight RCT studies, one utilized split-mouth design [36], and seven were parallel-arms [27, 28, 30–34]; two were multicenter RCTs [32, 33], two were sequential investigations on the same patients with different outcomes [28, 31].

**Table 2:** Characteristics of included studies

First author(year)	Study type	Patient		Graft		Surgery		Follow up (mouths)	Main outcomes	Radiographic
		Age (mean)	Gender (F/M)	Type	Name	Type of surgery	No.		histomorphometric	
Iezzi (2012)	CCT Non-randomized Controlled Trial	51–67(55)	6/9	BBX	Bio-Oss	MSFA	12	6	NFB, RBG, CT	NR
				PBX	Apatos Cortical		12			
J.-S. Lee (2017)	RCT Multicenter, Parallel-arms	49.17 ± 1.33	0/7	BBX	Bio-Oss	MSFA	7	6	NFB, RBG, CT	VR(cc), VHR
		42.14 ± 9.74	3/6	PBX	THE Graft		9			
H.-C. Lim (2017)	RCT Parallel-arms	51.07 ± 15.79	9/6	BBX	Bio-Oss Collagen	ARP	15	4	NR	VHR, HWR
		48.14 ± 16.11	11/3	PBX	Colla-Oss		14			
J.-S. Lee (2018)	RCT Parallel-arms	54.47 ± 11.00	15/32	BBX	Bio-Oss	ARP	47	4	NR	VR(%),VHR, HWR
		56.13 ± 10.20	15/32	PBX	THE Graft		47			
Koo (2020)	RCT Parallel-arms	34–81(54.29)	15/27	BBX	Bio-Oss	ARP	42	4	NFB, RBG, CT	NR
		31–82(55.82)	13/26	PBX	THE Graft		39			
Lai (2020)	RCT Parallel-arms	24–82(57)	25/13	BBX	Bio-Oss	ARP	17	18-20weeks	NFB, RBG, CT	NR
				PBX	ZcoreTM Porcine		21			
Galindo-Moreno (2022)	RCT split-mouth	36–73(56)	5/5	BBX	Bio-Oss + ACB	MSFA	10	6	NFB, RBG, CT	VR(cc), VHR
				PBX	Symbios^®^+ACB		10			
J.-H. Lee (2023)	retrospective case-control study	53.5 ± 11.2	30/37	BBX	Bio-Oss Collagen	ARP	67	6	NR	VHR, HWR
		53.6 ± 10.8	22/19	PBX	THE Graft Collagen		41			
Krennmair (2023)	RCT Parallel-arms	57.2 ± 7.3	8/5	BBX	Bio-Oss	MSFA	13	6	NFB, RBG, CT	VR(%),VHR
		58.3 ± 5.1	4/6	PBX	MinerOss^®^ XP		10			
Schmitt (2023)	RCT Multicenter, Parallel-arms	48.05 ± 13.52	7/2	BBX	Bio-Oss	MSFA	9	4–6	NR	VR(%)
		49.68 ± 16.93	9	PBX	The Graft		10			

**Abbreviations**: BBX, bovine bone xenograft; PBX, porcine bone xenograft; MSFA, maxillary sinus floor augmentation; ARP, alveolar ridge preservation; NFB, newly formed bone; RBG, residual bone graft; CT, connective tissue; VR, volume reduction; VHR, vertical height reduction; HWR, horizontal width reduction; NR, not reported; RCT, randomized controlled trial; CCT, case-controlled trial

All 10 studies used BBX from the same manufacturer. Among them, two studies utilized collagen-containing DBBM (Bio-Oss Collagen) in the BBX group, while the corresponding PBX groups also used collagen-containing DPBM (Colla-Oss [27] and THE Graft Collagen [29]). One study employed Bio-Oss with particle sizes of 1–2 mm in the BBX group, with the PBX group using equivalently sized particles (1–2 mm) of THE Graft [32]. The remaining seven studies compared Bio-Oss with particle sizes of 0.25–1.0 mm against PBX of the same particle size range from different manufacturers (1 with Apatois [35], 1 with Zcore [30], 1 with Symbios [36], 1 with MinerOss [34], and 3 with THE Graft [28, 31, 33]). For the PBX groups, although most studies used PBX from different brands, they shared similar chemical compositions as deproteinized porcine bone minerals (DPBM), thereby reducing heterogeneity in this study.

The selected studies reported healing times or time until implant placement between 4 and 6 months. Five studies reported healing times of 6 months [29, 33–36], 4 months in two sequential investigations studies [28, 31] and one study [27], 4–6 months in one study [32] and 18–20 weeks in one study [30]. In all the included studies, six studies collected samples through trephine biopsy during implant placement, which were then processed through standardized procedures and analyzed using histomorphometric techniques with the aid of optical microscopy combined with computer-based image analysis systems [30, 31, 33–36] The analysis evaluated the percentage of newly formed bone (NFB) and residual graft bone (RGB) relative to the total tissue, as well as the areas of connective tissue (CT). Meanwhile, seven studies conducted radiological assessments by obtaining CBCT scans immediately after bone grafting and performing another scan during the follow-up period, aligning and superimposing the 3D reconstructions based on anatomical landmarks to minimize positional differences [27–29, 32–34, 36]. The graft resorption indicators: volume changes were calculated using voxel subtraction (baseline vs. follow-up), while linear measurements (height/width reduction) were derived based on fixed reference points.

The histomorphometric and radiographic outcomes quantitatively included in the meta-analysis are detailed in Table 3. Subgroup classifications by surgical approach (MSFA or ARP) and follow-up duration are also included. For follow-up duration, studies were divided into two subgroups: long-term (≥ 5 months) and short-term (< 5 months). Specifically, the study by Schmitt et al. [32] (follow-up range: 4–6 months, mean ≈ 4.4 months) was categorized into the short-term subgroup based on its mean follow-up duration.

**Table 3:** Outcomes of included studies

First author(year)	Groupe Type	Follow-up Duration	Histomorphometric outcomes		CT(%)	Radiographic outcomes		VR
			NFB(%)	RBG(%)		VHR(mm)	HWR(mm)	
**MSFA**								
Iezzi (2012)	BBX	≥ 5	32.9(0.5)	32.8(2.1)	36.4(2.3)	NR	NR	NR
	PBX		31.8(2.9)	33.1(1.9)	38.7(2.7)	NR	NR	NR
J.-S. Lee (2017)	BBX	≥ 5	26.15(7.11)	25.74(18.75)	48.11(12.69)	-0.04(4.11)	NR	0.16(0.701)
	PBX		27.95(10.33)	15.4(8.53)	56.64(12.28)	0.35(2.2)	NR	0.11(1.05)
Galindo-Moreno (2022)	BBX	≥ 5	29.24(10.82)	31(17.29)	39.76(16.1)	-2.83(3.41)	NR	0.61(0.54)
	PBX		32.51(14.76)	31.21(19.61)	36.28(13.43)	-2.64(4.18)	NR	0.56(0.42)
Krennmair (2023)	BBX	≥ 5	22.9(5.1)	21.2(9.9)	56.1(9.5)	-1.5(2.39)	NR	8.5(6.3)
	PBX		27.7(5.6)	23.7(7.2)	47.5(9.5)	-1.2(2.55)	NR	7.5(5)
Schmitt (2023)	BBX	<5	NR	NR	NR	NR	NR	17.47(1132.45)
	PBX		NR	NR	NR	NR	NR	80.91(518.36)
**ARP**								
H.-C. Lim (2017)	BBX	<5	NR	NR	NR	-0.7(1.7)	-0.7(1.7)	NR
	PBX		NR	NR	NR	-1.1(1.3)	-1.1(1.3)	NR
J.-S. Lee (2018)	BBX	<5	NR	NR	NR	-1.45(1.92)	-1.59(2.34)	8.14(22.23)
	PBX		NR	NR	NR	-1.22(2.16)	-1.43(1.77)	6.48(24.23)
Koo (2020)	BBX	<5	15.07(10.52)	12.37(5.67)	72.56(10.07)	NR	NR	NR
	PBX		18.47(11.47)	12.21(5.75)	69.32(10.02)	NR	NR	NR
Lai (2020)	BBX	<5	36.21(26.51)	20.47(15.29)	43.32(15.78)	NR	NR	NR
	PBX		31.27(16.23)	19.52(9.19)	49.21(10.79)	NR	NR	NR
J.-H. Lee (2023)	BBX	≥ 5	NR	NR	NR	-1.03(1.95)	-1.38(2.2)	NR
	PBX		NR	NR	NR	-1.16(1.56)	-1.55(1.46)	NR

**Abbreviations**: BBX, bovine bone xenograft; PBX, porcine bone xenograft; MSFA, maxillary sinus floor augmentation; ARP, alveolar ridge preservation; NFB, newly formed bone; RBG, residual bone graft; CT, connective tissue; VR, volume reduction; VHR, vertical height reduction; HWR, horizontal width reduction; NR, not reported

### Risk of bias assessment

We assessed the risk of bias assessment for the ten included studies. The Newcastle-Ottawa Scale (NOS) was applied to the retrospective case-control study. The revised Cochrane Risk of Bias (ROB 2) tool was used to evaluated the quality of the eight RCTs, and one controlled clinical trial (CCT). Although primarily designed for RCTs, the ROB 2 tool is also applicable to CCTs due to the alignment of its domains and signaling questions.

**Fig. 2:**
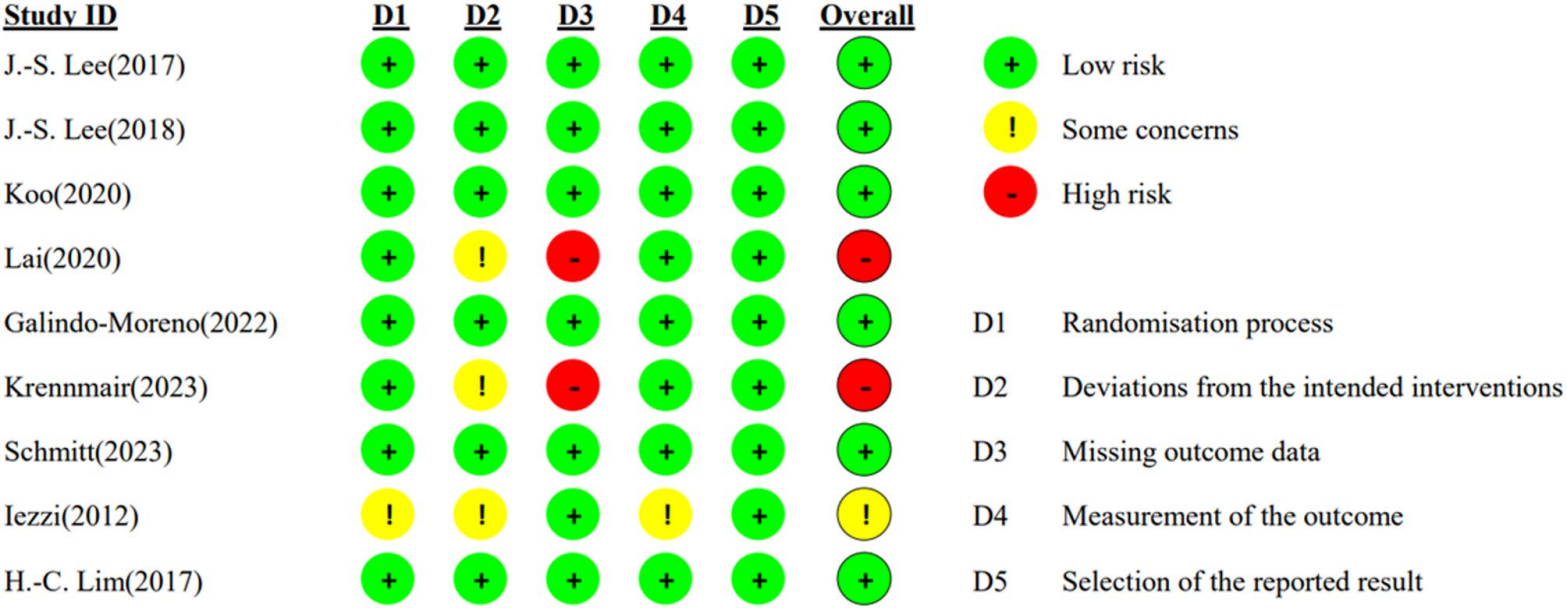
Risk of bias assessment of included studies according to the revised cochrane risk-of-bias tool for randomized trials (ROB 2)

In this Meta-analysis, one study was assessed using the NOS scale, scoring 7 out of 9, indicating a low risk of bias and reliable results [29]. Figure 2 depicts the risk of bias assessment of the included studies using the ROB 2 framework. Six studies showed an overall low risk of bias [27, 28, 31–33, 36]. Two RCTs showed an overall high risk of bias [30, 34]. This was due to a lack of data reporting and therefore a potential risk of reporting bias. The remaining one study showed an overall unclear risk of bias [35]. Some concerns were raised due to a lack of information on the randomization process, bias due to derivations from intended interventions, and bias due to using inappropriate methods to measure the outcome.

### Meta-analysis

#### Histomorphometric analysis

As shown in Table 3, among studies reporting histological outcomes, the categorization based on follow-up duration was aligned with that based on surgical approach; therefore, a unified subgroup analysis was conducted according to surgical approach.

#### NFB in grafted sites

**Fig. 3:**
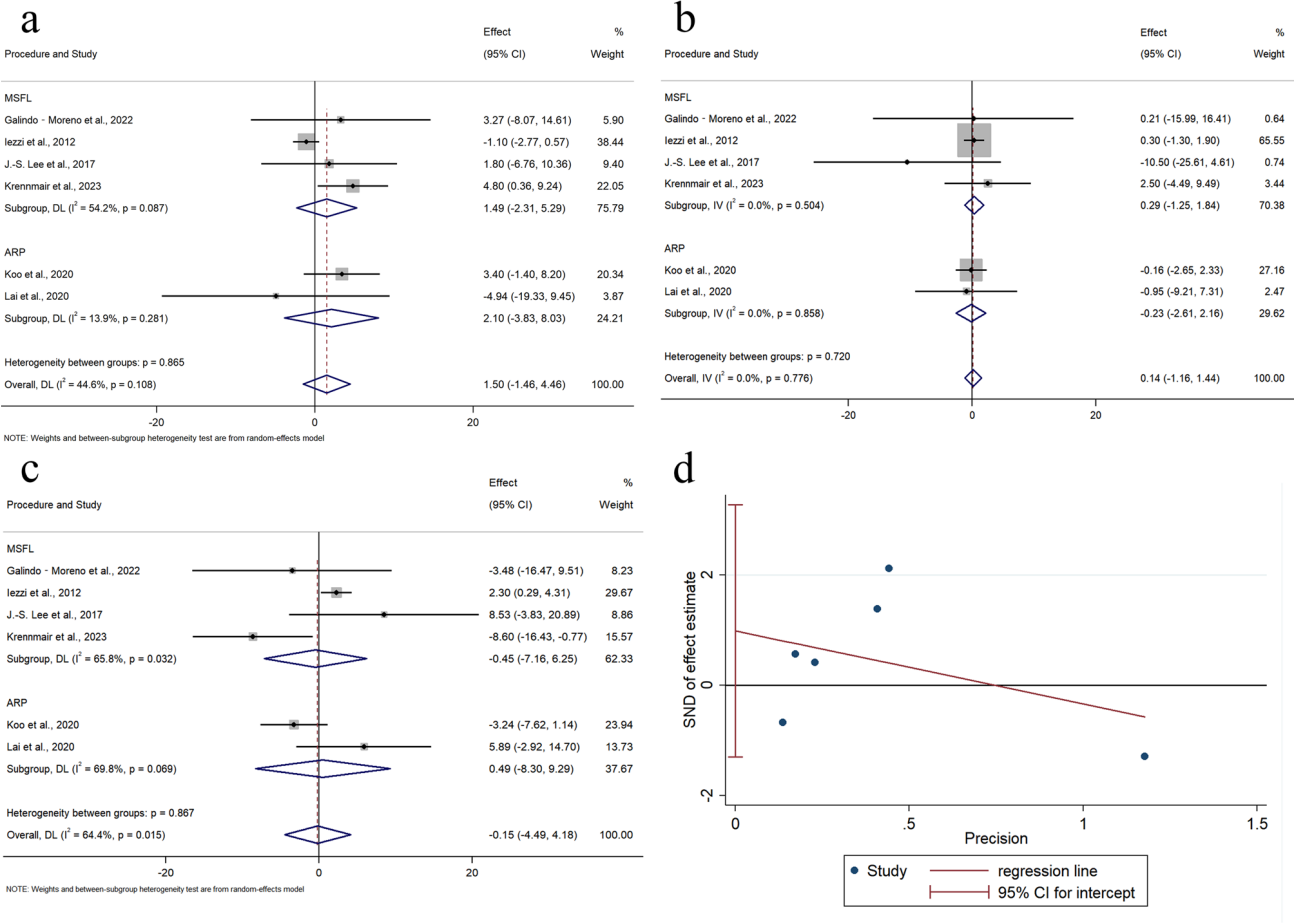
Meta-analysis on the Histomorphometric outcomes: **a** Forest plots of newly formed bone **b** Forest plots of residual bone graft **c** Forest plots of connective tissue **d** Egger’s test of newly formed bone

NFB was reported in six studies, involving 202 grafted sites (101 PBX, 101 BBX). Although the heterogeneity was moderate (I² = 44.6%, *p* = 0.108), a random effects model was applied to account for potential clinical and methodological differences across studies. As shown in Fig. 3a, there was no significant difference in NFB between PBX and BBX (WMD = 1.5, 95% CI: − 1.46 to 4.46, *p* = 0.321). Subgroup analyses based on surgical type (MSFA or ARP) also showed no significant differences. Despite the limited number of studies, we conducted a test for publication bias. Using the Egger test, we did not find evidence of significant publication bias (*P*>0.05; Fig. 3d).

#### RBG in grafted sites

RBG was reported in six studies, involving 202 grafted sites (101 PBX, 101 BBX). A fixed-effects model was applied (I^2^ = 0%, *p* = 0.776). As shown in Fig. 3b, no significant difference in RBG percentage was observed between the studies (WMD = 0.14, 95% CI: − 1.16 to 1.44, *p* = 0.833). Subgroup analyses based on surgical type (MSFA or ARP) also showed no statistically significant differences.

#### CT in grafted sites

CT was reported in six studies, involving 202 sites (101 PBX, 101 BBX). A random-effects model was used due to significant heterogeneity (I² = 64.4%, *p* = 0.015). As shown in Fig. 3c, no significant difference in CT percentage was observed (WMD = − 0.15, 95% CI: − 4.49 to 4.18; *p* = 0.944). Subgroup analyses based on surgical type (MSFA or ARP) also showed no statistically significant differences.

### Radiographic analysis

#### VHR in grafted sites

**Fig. 4:**
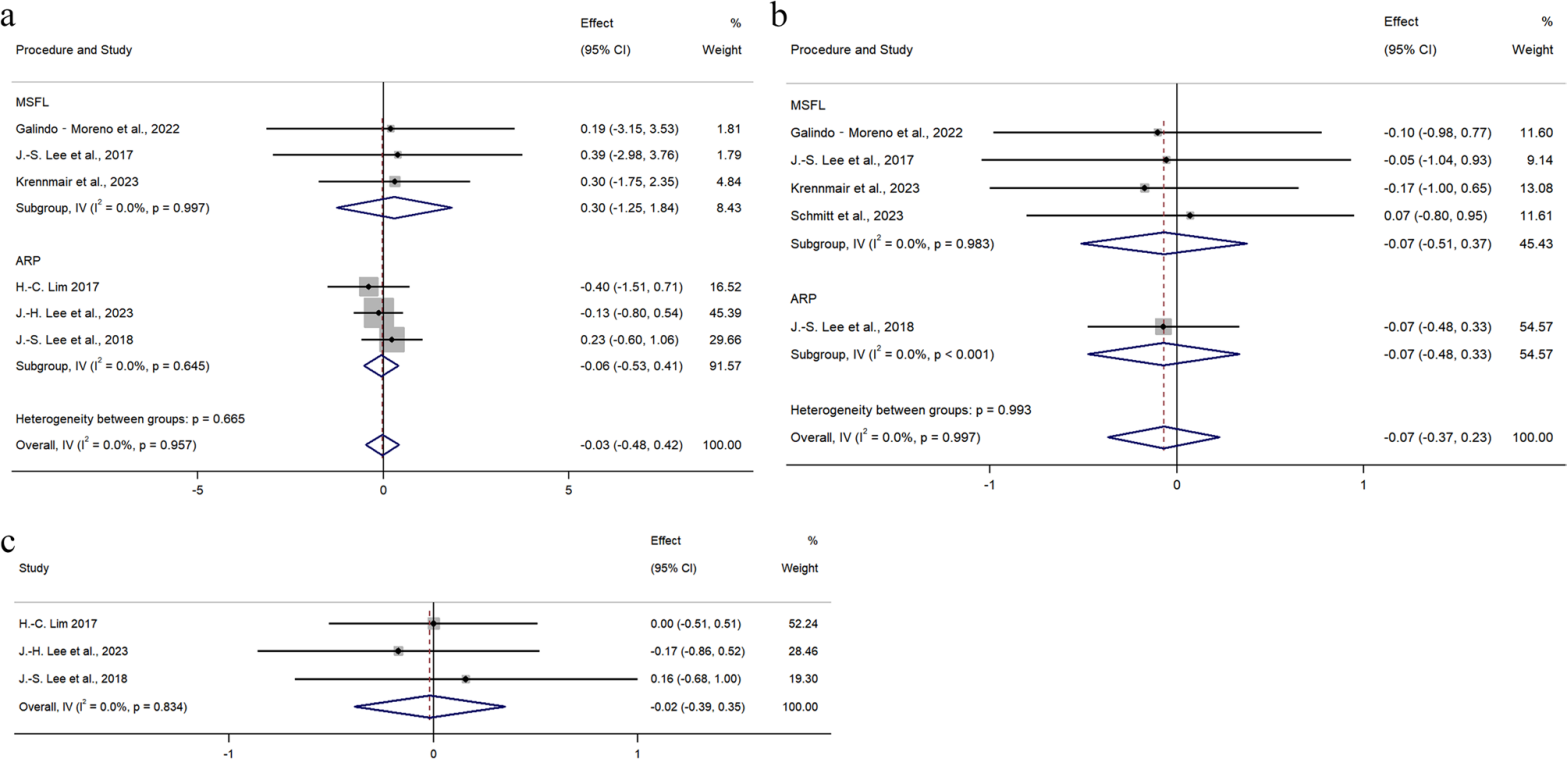
Meta-analysis on the Radiographic outcomes: **a** Forest plots of vertical height reduction **b** Forest plots of volume reduction **c** Forest plots of horizontal width reduction

**Fig. 5:**
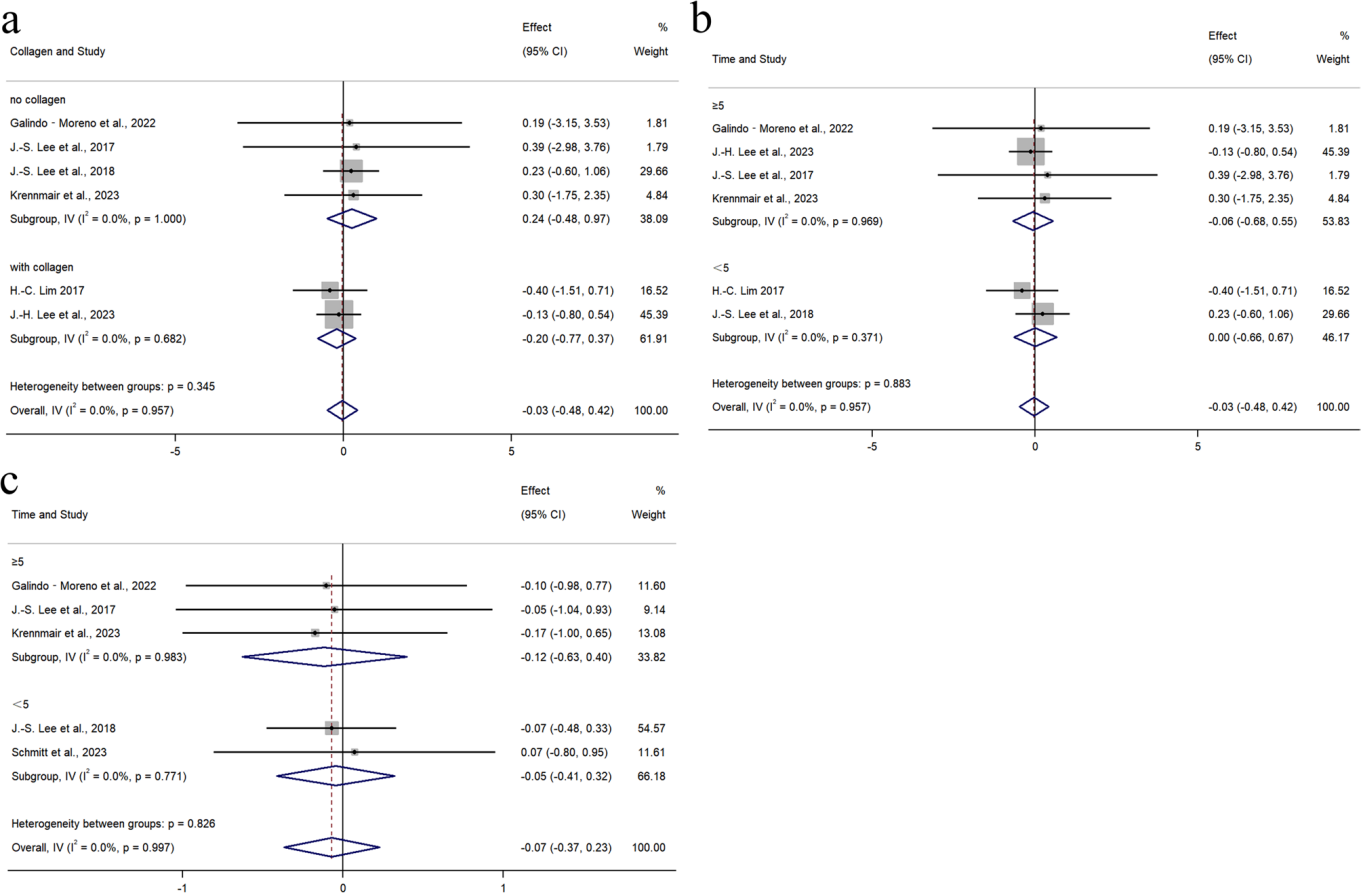
Forest plots of meta-analyses for **a** vertical height reduction levels stratified by collagen **b** vertical height reduction levels stratified by healing time **c** volume reduction levels stratified by healing time

VHR was reported in six studies, involving 290 sites (132 PBX, 158 BBX). A fixed-effects model was used (I² = 0%, *p* = 0.957). As shown in Fig. 4a, no significant difference in VHR was observed between the groups (WMD = − 0.03, 95% CI: −0.48 to 0.42; *p* = 0.899). Subgroup analyses based on surgical type (MSFA or ARP) and follow-up duration (≥ 5 years or < 5 years) showed no statistically significant differences (Fig. 5a). Additionally, two studies utilized composite materials containing collagen components. Subgroup analysis based on material composition (no collagen or with collagen) also revealed no statistically significant differences (Fig. 5b).

#### VR of grafts

VR was reported in five studies, with 173 sites evaluated (86 PBX, 87 BBX). Due to the absence of heterogeneity (I² = 0%, *p* = 0.997), a fixed-effects model was applied. Standardized mean difference (SMD) was used to account for differences in measurement units across studies. To normalize the VR data and eliminate the influence of different measurement units across studies, we used the Standardized Mean Difference (SMD) as the effect size measure. As depicted in Fig. 4b, no significant difference in VR was found between the two groups (SMD = − 0.07, 95% CI: −0.37 to 0.23; *p* = 0.645). Subgroup analyses based on surgical type (MSFA or ARP) and follow-up duration (≥ 5 years or < 5 years) showed no statistically significant differences (Fig. 5c).

#### HWR in grafted sites

HWR was mentioned in three studies, involving 231 sites (103 PBX, 128 BBX). A fixed-effects model was applied (I² = 0%, *p* = 0.834). As shown in Fig. 4c, no significant difference in HWR was observed (WMD = − 0.02, 95% CI: −0.39 to 0.35; *p* = 0.926). Since all three studies exclusively utilized the ARP surgical approach, and the limited number of eligible studies (*n* = 3) precluded subgroup comparisons, no subgroup analyses were performed based on surgical approach or follow-up duration.

### Sensitivity analyses

**Fig. 6:**
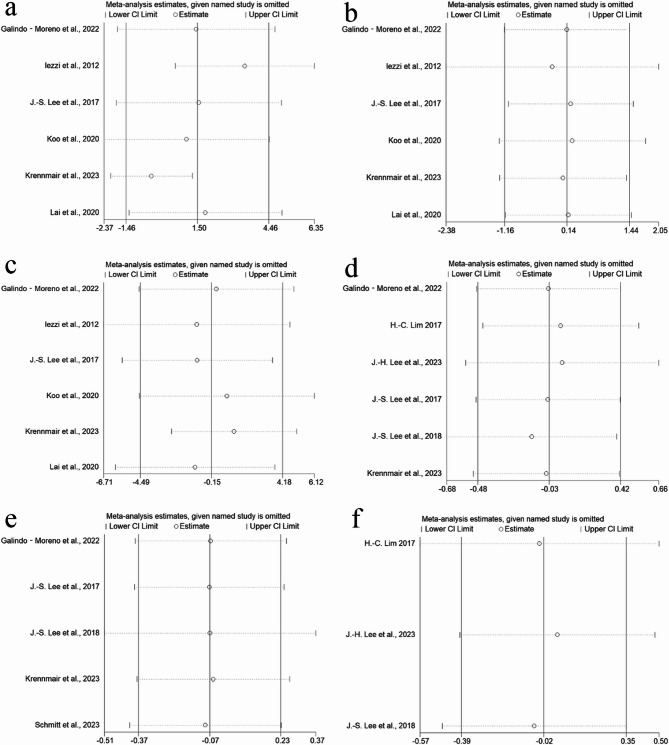
Sensitivity analyses **a** newly formed bone **b** residual bone graft **c** connective tissue **d** vertical height reduction **e** volume reduction **f** horizontal width reduction

Sensitivity analyses were performed for both primary and all secondary outcomes. For the primary outcome, exclusion of the study by Iezzi et al. resulted in a significant change in the pooled effect size, suggesting its potential influence on the overall findings (Fig. 6a). However, removal of other individual studies did not significantly alter the pooled effect estimates, indicating relative robustness of the meta-analysis results. Among the five secondary outcomes, sequential exclusion of each study did not lead to significant changes in the pooled effect sizes, further supporting the stability of the meta-analysis findings (Fig. 6b-f).

## Discussion

This study compared the histomorphometric and radiographic outcomes of PBX and BBX in MSFA and ARP. PBX exhibited no notable differences from BBX in histomorphometric and radiographic outcomes. Both groups demonstrated comparable outcomes in terms of the NFB, RBG and CT, as well as VHR, HWR, and VR of graft material.

Recently, several systematic reviews have highlighted the effectiveness of various grafting materials in increasing bone volume in the floor of the maxillary sinus [37, 38] and reducing bone loss post-extraction [39]. Bio-Oss (a BBX) is one of the most commonly used biomaterials in dental applications due to its crystallinity and physicochemical properties closely resembling human cancellous bone. Acting as an osteoconductive scaffold, BBX supports osteoblast migration from the sinus wall [40, 41]. Similarly, PBX, which mimics human bone structure, has shown promising results in dental applications [20, 42]. Animal studies have shown that BBX and PBX yield similar histological outcomes, such as bone formation area, osteogenic potential, and gene expression [43]. Long-term clinical studies further support the comparable biocompatibility of PBX to BBX [44, 45]. In addition, PBX offers distinct advantages in cases where patients have concerns about the religious acceptability of bovine-derived materials or the potential risks associated with prion diseases [15, 18]. In such scenarios, PBX serves as a viable alternative to BBX, delivering comparable clinical outcomes. Understanding the clinical equivalence of these materials is crucial for broadening treatment options and addressing diverse patient needs.

All included studies in this paper employed standardized histomorphometric and radiographic assessment protocols, albeit with minor variations in technical specifications. For histomorphometric analysis, bone samples were obtained via trephine biopsy during implant surgery, followed by sequential processing including fixation, decalcification, embedding, sectioning, and staining. Quantitative assessments were completed using optical microscopy combined with computer-based image analysis systems. Radiographic evaluation involved cone-beam computed tomography (CBCT) scans performed immediately after bone grafting and at 4–6 month follow-ups. Three-dimensional reconstructions were generated, and images from both timepoints were superimposed based on anatomical landmarks using specialized software to measure radiological changes in bone graft materials. While slight differences existed in operational workflows, the implementation protocols across all studies remain methodologically valid. Such discrepancies inevitably arise from inherent variations in materials, equipment, and software among different research settings. Crucially, as our assessment targets a complex multifactorial biological process, the standardization of experimental methodologies and evaluation metrics holds greater scientific significance than minor technical variances.

This meta-analysis demonstrates the clinical equivalence of PBX and BBX in bone grafting, consistent with findings from previous studies. Most studies reported no significant differences in histomorphometric and radiographic outcomes, confirming the reliability of both materials. However, one study observed slightly higher NFB and lower CT in the PBX group [34], suggesting a potential material-specific effect.

One study by Iezzi, Degidi [35] stood out due to its crossover design, involving 15 patients who underwent 30 sinus augmentations using five different biomaterials, yielding a total of 60 bone cores. This design minimized inter-patient variability and thus carried greater weight in the overall analysis. Additionally, Iezzi et al. provided direct comparative data on PBX and BBX in MSFA, which are highly relevant to our study. Although excluding this study in a sensitivity analysis yielded a statistically significant difference in NFB between PBX and BBX, we deemed its inclusion justified given the robust design. Interestingly, the sensitivity analysis also hinted at a potential advantage of PBX in promoting new bone formation. This finding warrants further investigation to explore its underlying mechanisms and potential advantages in specific clinical scenarios, particularly for patients requiring tailored treatment approaches.

Notably, one study did not directly compare PBX and BBX alone, but mixed autologous cortical bone (ACB) in a 20:80 volume ratio with each xenograft [36]. Their analysis specifically examined cellularity, microvascular density, and protein expression. Immunohistochemical results showed no significant differences between sites grafted with BBX + ACB and PBX + ACB, except for leukocytes, osteocytes, and vessels, which were more abundant in the BBX + ACB group. Despite the mixing protocol, a paired meta-analysis indicated that the combination of allograft and autograft did not provide a significant advantage for newly formed bone, aligning with our broader finding that PBX and BBX demonstrate comparable outcomes [46].

Additionally, Galindo-Moreno, Abril‐García [36] also investigated the gene expression related to bone formation and maintenance of homeostasis, especially those associated with the osteo-differentiation pathway in mesenchymal stromal cells. They noted that two earlier studies [47, 48] reported few statistical differences between the groups, aligning with their own findings. However, they found a significant difference in alkaline phosphatase (ALP) gene expression, which was greater in the PBX + ACB group than in the BBX + ACB group [36]. In mineralized tissue cells, ALP is highly expressed and plays a crucial role in hard tissue formation [49]. The BMP/RUNX2/Osterix axis and the WNT signaling pathway, which closely interact, are the primary regulators of ALP expression [50]. Therefore, it is suggested that future studies could investigate the specific mechanisms of higher ALP expression in the porcine-derived xenograft material.

One notable limitation in this meta-analysis stems from variation in healing periods among the included studies (4–6 months). To evaluate its potential impact, we categorized trials based on healing duration (≥ 5 months versus < 5 months) and analyzed outcomes between subgroups. No statistically meaningful differences emerged between groups, implying that the efficacy comparison of porcine and bovine xenografts might remain stable throughout this temporal spectrum. Current interpretations require caution, as this statistical non-significance could alternatively stem from insufficient analytical power—possibly related to inadequate subgroup sample sizes. Future recommendations include prospective studies with standardized healing intervals to further validate these findings, as well as longer-term studies (> 6 months) to evaluate late remodeling differences.

It is important to note that we included a retrospective case-control study [29], as its methodology aligned with the inclusion criteria and held scientific relevance. This study provided direct comparative data between PBX and BBX under uniform surgical protocols (e.g., identical graft placement techniques, follow-up timelines, and radiographic evaluation methods), which reduced confounders from procedural variability and adhered to the requirement for direct comparisons between the two biomaterials, making it highly relevant to our research. Furthermore, sensitivity analyses excluding this retrospective case-control study demonstrated robust effect estimates, further supporting the rationale for including Lee et al.‘s work. Additionally, like another study in our analysis, this research evaluated collagen-enriched xenografts, whereas other studies compared PBX and BBX without collagen. We therefore conducted subgroup analyses stratified by collagen composition (no collagen or with collagen), which revealed no significant differences between subgroups at the radiographic level. To address potential heterogeneity introduced by material composition—particularly variations in PBX manufacturers across studies—we performed sensitivity analyses for each outcome discussed (NFB, RGB, CT, VHR, HWR, VR). The results indicated stable conclusions even after excluding individual studies, enhancing confidence in the reliability of our findings. Multiple studies have reported that their xenograft particles exhibited histomorphometric outcomes comparable to both the standard benchmark (inorganic bovine bone) and other porcine-derived biomaterials in clinical practice [34, 36]. Network meta-analyses also showed no significant differences in direct comparisons of bone substitute biomaterials from the same source [51]. However, one study comparing two BBX grafts identified differences in physicochemical properties that may influence clinical outcomes [52]. Similarly, another study suggested variations in biomaterial production methods could lead to distinct biological responses [53]. Based on these findings, differences in materials, including variations in production methods and physicochemical properties, may contribute to heterogeneity in research outcomes and should be carefully considered in future studies.

The present study has certain limitations. First, the bone grafting materials were obtained from different manufacturers, introducing potential variability. Second, the limited number of RCTs and small sample sizes in the included studies constrain the robustness of the conclusions. Third, some studies did not provide detailed data on confounding factors, such as smoking, diabetes, and brushing habits, which may have affected the risk of bone graft failure. Fourth, while we evaluated histomorphometric and radiographic outcomes, the included literature lacked assessments of clinically relevant patient-centered metrics (e.g., post-operative pain, aesthetic satisfaction) and sociocultural influences on graft selection (e.g., religious prohibitions in Muslim/Hindu populations) [17, 54].

Although bovine-derived xenografts (BBX) remain predominant in clinical practice, our analysis demonstrates that porcine-derived xenografts (PBX) achieve equivalent performance in bone regeneration and radiographic stability. These findings suggest PBX can serve as a clinically comparable alternative to BBX in populations without cultural or religious restrictions on porcine-derived materials. It should be noted that although both materials are clinically feasible, their biological equivalence does not imply interchangeability in practical applications. Religious doctrines and cultural norms regarding taboos on pig or cow products may profoundly influence patient acceptance, regardless of technical success. Therefore, future research can focus more on qualitative studies to explore factors such as religion, culture, and patient preferences in xenotransplantation choices. This will help develop evidence-based and patient-centered transplantation strategies that are more in line with socio-cultural contexts.

## Conclusions

The current systematic review found that both PBX and BBX achieved similar histomorphometric and radiographic outcomes in bone grafting procedures. These findings support PBX as a clinically viable alternative to BBX, particularly in populations without cultural or religious restrictions on porcine-derived materials. However, further high-quality RCTs with sufficiently powered sample sizes are needed to validate these results. Future studies should compare the clinical indicators of PBX and BBX over extended follow-up periods to explore the differences in their long-term effects, such as implant survival, graft resorption rates, and the stability of bone formation area over time, thereby identifying which material may offer superior clinical outcomes in bone grafting.

## Data Availability

The data that support the findings of this study are available from the corresponding author, upon reasonable request.

## Abbreviations

PBX: Porcine bone xenografts
BBX: Bovine bone xenografts
MSFA: Maxillary sinus floor augmentation
ARP: Alveolar ridge preservation
NFB: Newly formed bone
RBG: Residual bone graft
CT: Connective tissue
VHR: Vertical height reduction
HWR: Horizontal width reduction
VR: Volume reduction
ALP: Alkaline phosphatase
ACB: Autologous cortical bone
